# Does the trematode *Centrocestus formosanus* affect the locomotory activity of the mollusc *Melanoides tuberculatus*?

**DOI:** 10.1186/1756-3305-6-92

**Published:** 2013-04-10

**Authors:** Everton Gustavo Nunes dos Santos, Viviane da Silva Costa, Cláudia Portes Santos

**Affiliations:** 1 Laboratório de Avaliação e Promoção da Saúde Ambiental, Instituto Oswaldo Cruz, Fundação Oswaldo Cruz, Av. Brasil, 4.365, Manguinhos, Rio de Janeiro 21040-360, Brazil

**Keywords:** Behavioural changes, Movement, Locomotory activity, Trematoda, Mollusca, Brazil

## Abstract

**Background:**

*Melanoides tuberculatus* (Müller, 1774) (Thiaridae), an introduced gastropod mollusc with a wide geographical distribution in the Neotropics, is the intermediate host of the trematode *Centrocestus formosanus* (Nishigori, 1924) (Heterophyidae). This parasite is considered to be pathogenic to humans. The aim of the present work was to evaluate the locomotory activity of uninfected *M*. *tuberculatus* compared with those naturally infected with *C*. *formosanus*.

**Findings:**

The locomotory activity of each mollusc was recorded using an image analysis biomonitoring system, Videomex-V ®, to evaluate and quantify the parameters of ‘Stereotypic’ and ‘Resting time’. The Generalized Estimating Equation analysis of locomotory activity of *M*. *tuberculatus* infected with *C*. *formosanus* revealed significant differences compared with uninfected molluscs for the parameters ‘Stereotypic time’ and ‘Resting time’ with a reduction of movement. The variations in the values of the monitoring intervals recorded showed a significant difference for the infected molluscs in the case of Stereotypic time, with an irregular locomotory activity pattern, as compared to that of uninfected molluscs. The analysis of the standard length of all molluscs did not exhibit any correlation with locomotory activity, showing that *C*. *formosanus* is able to alter the locomotory activity of its snail host regardless of the standard length.

**Conclusions:**

The trematode *C*. *formosanus* affects the locomotory activity of the mollusc *M*. *tuberculatus* by reducing its movement and causing it to exhibit an irregular pattern of activity, both of which are independent of the snail's standard length.

## Findings

### Background

Parasitic infections can alter the physiology and behaviour of their hosts and in an ecological context, such changes can increase the exposure of the host to various predators [1]. For instance, parasites that use molluscs as intermediate hosts can, during the process of asexual reproduction, utilize the host`s resources, causing an immediate effect on its physiology [2]. Accordingly, Levri et al. [3] showed that the intramolluscan stage of the trematode *Microphallus* sp. is able to alter the rate of both the movement in response to light and the vertical movement of the mollusc *Potamopyrgus antipodarum* (Gray, 1853), possibly enhancing its chances of transmission to the next host and decreasing the probability of the latter being captured by predators.

Aquatic environments offer ideal conditions for the development and maintenance of parasite life cycles, mainly because the water viscosity facilitates the dispersal of eggs and free-living stages, enabling the maintenance of complex parasite life cycles [4]. In the area of Rio de Janeiro, Brazil, the life cycles of digenean species have been elucidated in the ecosystem of the Rodrigo de Freitas lagoon [5-8] and maintained in our laboratory, enabling us to compare the swimming behaviour of an intermediate fish host before and after a digenean infection using an image analysis biomonitoring system [9]. Although the avian and fish fauna of the lagoon are well known, there are few studies on molluscs in this area [6], among which is the ‘red-rim melania’ *Melanoides tuberculatus* (Müller, 1774) (Thiaridae), an introduced gastropod snail which now has a wide geographical distribution throughout the Neotropics [10].

*Melanoides tuberculatus* is reported to be a well-adapted intermediate host for members of at least 16 trematode families, including 25 genera and 37 species [11]. Among these, the trematode *Centrocestus formosanus* (Nishigori 1924), parasitic as an adult in mammals and birds, is considered to be pathogenic to humans; it is also able to cause the death of small fish, as a result of a massive infection of metacercariae [12-14]. Although it’s negative impact on fish intermediate hosts and humans is well known [13,14], the effect of the parasitism of *C*. *formosanus* on *M*. *tuberculatus* requires further investigation.

The aim of this study was to evaluate whether *C*. *formosanus* can alter the locomotory activity of *M*. *tuberculatus* by comparing individual molluscs, which were naturally parasitized with uninfected ones using an image analysis biomonitoring system.

### Methods

#### Collection and analysis of M. tuberculatus

A total of 65 specimens of *M*. *tuberculatus* (15–25 mm standard length) were randomly collected at a single station on the edge of the Rodrigo de Freitas Lagoon, Rio de Janeiro, Brazil (22°97’00”S, 43°21’65”W). They were maintained alive in laboratory aquaria filled with filtered lagoon water and fed with commercial fish food flakes. During a period of 3 months, the molluscs were examined for cercariae once per week. Individual snails were isolated in 20 mL vessels containing filtered lagoon water, exposed to artificial light for 4 hours and examined under a stereomicroscope. All shed cercariae were identified as *C*. *formosanus* following Scholz and Salgado-Maldonado (2000). Thus, the molluscs were separated into two groups: uninfected and infected. After the experiments, all molluscs were examined to confirm the presence or absence of parasites.

#### Image analysis biomonitoring system

The image analysis biomonitoring system (IABS) used in this study is that used by Magalhães et al. [9,15]. The IABS is composed of an illuminating cabin and a recording cabin made of acrylate and holding a fixed analogue video camera. The images were sent to a Videomex V® video tracking system (Columbus Instruments, Ohio, USA) and analyzed using the software Travelled Distance of Multiple Objects (TDMO). Data were sent to a computer and recorded in an Excel spreadsheet.

During the experiments, molluscs were placed individually in 8 holding boxes (4 × 4 × 2 cm each) made of opaque acrylate with 3 mm holes, which were kept inside an opaque glass aquarium of 30 L capacity (35 × 35 × 25 cm) filled with filtered water from the lagoon. Submerged water pumps maintained the water circulation under a controlled temperature of 21°C.

Each experiment was performed during a 68 min period, with 20 min of acclimation and 48 min of monitoring analysis recorded at 48 intervals of 1 min each. All values of each interval of ‘Stereotypic time’ and ‘Resting time’ were tested simultaneously and used for statistical analysis. ‘Stereotypic time’ is the total number of seconds during the interval in which the mollusc performed some movement activity, whereas ‘Resting time’ is the total number of seconds during the interval spent without movement.

#### Statistical analysis

All statistical analyses were performed using the R statistical program (version 2.15.0) [16]. The Mann–Whitney test was used to evaluate the differences between the standard length of infected and uninfected molluscs. This nonparametric test was used due to the non-normal distribution of the samples.

For the behavioural experiments, we used the Generalized Estimating Equation (GEE) with the packages “geepack” and “yags” [17]. This test analyzed repeat measurements of the locomotory activity data (‘Stereotypic time’ and ‘Resting time’) of infected and uninfected molluscs, estimating the parameters of regression and variance in the dependent timings. In an attempt to verify a locomotory pattern in each of the groups (uninfected and infected molluscs), the differences between the values obtained in each recorded interval during the monitoring period were analyzed using GEE. In addition, also using GEE, we tested the influence of the standard length of infected and uninfected molluscs in relation to the ‘Stereotypic time’ and ‘Resting time’.

The model was performed using each behavioural parameter as a variable response with a Gaussian error distribution, identity-link function and exchangeable correlation structure. A goodness-of-fit statistic, the quasi-likelihood information criterion (QIC), was used for evaluating the models [18]. The level of significance assumed for statistical tests was 5%.

#### Ethical considerations

This research was licensed by the Brazilian Institute of Environment and Renewable Natural Resources (IBAMA no. 15898–1) and approved by the Animal Ethics Committee of the Oswaldo Cruz Foundation (CEUA-FIOCRUZ LW-12/10), in accordance with the guidelines of the Brazilian College for Animal Experiments (COBEA).

### Results

The GEE analysis of the standard length of all 65 molluscs (15–25 mm, mean 18.55 ± 1.9) did not exhibit any correlation with locomotory activity (Stereotypic time, coefficient estimation = −0.48 ± 1.11, Wald statistic_1584_ = 0.19, *P* = 0.66; Resting time, coefficient estimation = 0.58 ± 1.19, Wald statistic_1584_ = 0.24, *P* = 0.62).

Of the molluscs analyzed, 30 did not shed cercariae and 35 shed cercariae of *C*. *formosanus* (only) daily. The uninfected molluscs measured 15–20mm (18 ± 1.3) in standard length and the infected ones were 15–25mm (19 ± 2.3). The Mann–Whitney *U* test showed significant differences in standard length between the infected and uninfected molluscs (*W*_64_ =1103, *P* < 0.001).

**Figure 1:**
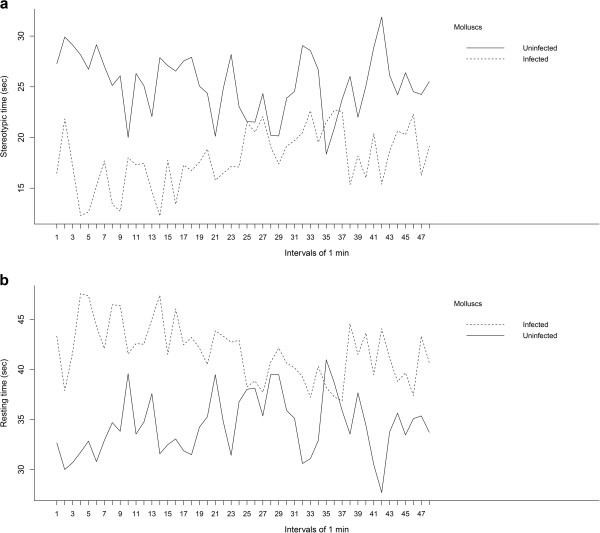
**Monitoring analysis of Melanoides tuberculatus uninfected and naturally infected by Centrocestus formosanus.** Comparative graphics showing significant differences between 48 intervals of 1 min of monitoring analysis of **(a)** Stereotypic time (seconds) and **(b)** Resting time (seconds) of *Melanoides tuberculatus* uninfected and naturally infected by *Centrocestus formosanus*.

The GEE analysis of locomotory activity revealed significant differences between infected and uninfected molluscs for the parameters ‘Stereotypic time’ (coefficient estimation = 10.50 ± 4.07, Wald statistic_3312_ = 6.66, *P* = 0.009, Figure 1a) and ‘Resting time’ (coefficient estimation = −10.31 ± 4.14, Wald statistic_3312_ = 6.19, *P* = 0.01, Figure 1b).

The GEE analysis between the values of the 48 monitoring intervals recorded during the monitoring period compared the locomotory pattern in each of the groups (uninfected and infected molluscs). The differences in the values for the uninfected molluscs, was not significant for the parameters Stereotypic time (coefficient estimation = −0.04 ± 0.08, Wald statistic_3312_ = 0.26, *P* = 0.60) and Resting time (coefficient estimation = 0.04 ± 0.89, Wald statistic_3312_ = 0.21, *P* = 0.64); this was thus considered a regular pattern. However, the same test for the infected molluscs showed a marginally significant difference for Resting time (coefficient estimation = −0.10 ± 0.05, Wald statistic_3312_ = 3.81, *P* = 0.05) and a significant difference in the case of Stereotypic time (coefficient estimation = 0.10 ± 0.05, Wald statistic_3312_ = 3.91, *P* = 0.04); this was considered an irregular pattern.

### Discussion

Host-parasite relationships are an intriguing subject as they involve, for example, competition for energy and nutrients involving complex physiological processes, as well as changes in the rate of growth and other metabolic variation [19-21].

Stress conditions are reported to occur due to metabolic changes in molluscan hosts caused by trematodes when the intramolluscan stages influence the mobilization of calcium between the shell and haemolymph during their larval development [21]. In addition, the accelerated metabolic rate of energy of the infected molluscs caused by the development of parasite larvae results in a negative imbalance due to the high consumption of nutrients [20-22]. Thus, a consequence of such effects can probably alter the behavioural pattern of the molluscan host.

The dimensions of the shells of molluscs may also be influenced by parasitism [23-25]. In our study, infected *M*. *tuberculatus* were significantly longer than the uninfected ones (*P* < 0.001). However, a comparison of the length of all molluscs with their locomotory activity showed no significant correlation. Nevertheless, when the locomotory activity of infected vs. uninfected hosts was compared, the former had a significantly reduced ‘Stereotypic time’ and an increased ‘Resting time’. Although Miller & Poulin [2] have reported that parasitized snails had larger dimensions of shells and were found to move a significantly shorter distance than uninfected snails, our survey shows that the parasite *C*. *formosanus* is able to alter the locomotory activity of *M*. *tuberculatus* irrespective of the snail’s standard length.

The host-parasite association can be seen as a constant conflict between the needs of the parasite and those of the host for growth, reproduction and survival [26]. Consequently, it is possible that the parasite influences the behaviour of its hosts in ways that increase the probability of its transmission, as discussed by [27]. *Melanoides tuberculatus* infected with *Centrocestus formosanus*, in addition to a reduced movement, exhibited an irregular locomotory activity pattern, whereas uninfected snails had a regular pattern of locomotory activity during the biomonitoring period. A similar experiment by Santos et al. [9], using a Videomex V® system, assessed the locomotory activity of fish hosts in the same lagoon, and these authors suggested that a lethargic and less motile parasitized fish is more likely to be preyed upon by the definitive host. In the case of the less motile snail host, we consider that it is more likely to influence the life cycle of *C*. *formosanus* by concentrating the shedding of cercariae into a more restricted area.

### Conclusions

The trematode *Centrocestus formosanus* affects the locomotory activity of the mollusc *Melanoides tuberculatus* by reducing its movement and causing it to exhibit an irregular pattern of activity, both of which are independent of the snail's standard length.

## Competing interests

The authors declare that they have no competing interests.

## Authors’ contributions

CPS and EGNS conceived and designed the study. VSC performed the parasitological examinations. EGNS performed the tests and did the statistical analysis. CPS and EGNS wrote the paper. Supervisor of MSc: CPS. All authors read and approved the final version of the manuscript.
